# Efficient synthesis, biological evaluation, and docking study of isatin based derivatives as caspase inhibitors

**DOI:** 10.1080/14756366.2020.1809388

**Published:** 2020-08-25

**Authors:** Loghman Firoozpour, Lixin Gao, Setareh Moghimi, Parvin Pasalar, Jamshid Davoodi, Ming-Wei Wang, Zahra Rezaei, Armin Dadgar, Hoda Yahyavi, Massoud Amanlou, Alireza Foroumadi

**Affiliations:** a Drug Design and Development Research Center, The Institute of Pharmaceutical Sciences (TIPS), Tehran University of Medical Sciences, Tehran, Iran; b National Center for Drug Screening, Shanghai Institute of Materia Medica, Chinese Academy of Sciences, Shanghai, China; c Department of Biochemistry, Faculty of Medicine, Tehran University of Medical Sciences, Tehran, Iran; d Institute of Biochemistry and Biophysics, University of Tehran, Tehran, Iran; e Department of Medicinal Chemistry, Faculty of Pharmacy, Tehran University of Medical Sciences, Tehran, Iran; f Pharmaceutical Sciences Research Center, Kermanshah University of Medical Sciences, Kermanshah, Iran

**Keywords:** Caspase inhibitor, Isatin sulphonamides, docking studies, Pharmacophore, apoptosis

## Abstract

In this paper, a new series of isatin-sulphonamide based derivatives were designed, synthesised and evaluated as caspase inhibitors. The compounds containing 1-(pyrrolidinyl)sulphonyl and 2-(phenoxymethyl)pyrrolidin-1-yl)sulphonyl substitution at C5 position of isatin core exhibited better results compared to unsubstituted derivatives. According to the results of caspase inhibitory activity, compound **20d** showed moderate inhibitory activity against caspase-3 and −7 *in vitro* compared to Ac-DEVD-CHO (IC_50_ = 0.016 ± 0.002 μM). Among the studied compounds, some active inhibitors with IC_50s_ in the range of 2.33–116.91 μM were identified. The activity of compound **20d** was rationalised by the molecular modelling studies exhibiting the additional van der Waals interaction of N-phenylacetamide substitution along with efficacious T-shaped π-π and pi-cation interactions. The introduction of compound **20d** with good caspase inhibitory activity will help researchers to find more potent agents.

## Introduction

Caspases, cysteinyl aspartate-specific proteases, are a family of signalling molecules playing a key role in apoptosis. Apoptosis is a physiological suicide process which gives an opportunity to dismantle unwanted cells population during animal development and tissue homeostasis^1^. Morphological changes such as DNA strand breaks along with nuclear membrane damage occur as a result of some biochemical events during apoptosis^2^. Two intrinsic and extrinsic pathways are responsible for initiating the apoptosis process. Binding of certain protein to the death receptor activates caspase-8 and subsequently triggers apoptosis by promoting effector caspases (–3, −6, −7). It should be noted that caspase enzymes are classified as initiator (caspase-2, caspase-8, caspase-9, and caspase-10) and effector (caspase-3, caspase-6, and caspase-7) which are exploited in re sponse to proapoptotic signals^3^^,^^4^. Caspase-3, activated by the upstream caspase-8 and caspase-9, is considered as a crucial mediator of apoptotic cell death in mammals by which more than 500 cellular substrates are cleaved to execute the apoptosis programme^5^^,^^6^. Regarding the close relationship between apoptosis and the wide range of disease, caspase inhibitors are capable of opening new paths to treat several diseases involving immunodeficiency, Alzheimer’s, Parkinson’s, Huntington’s diseases, ischaemia, brain trauma, and amyotrophic lateral sclerosis^7^. Taking the obtained data from the X-ray structure of caspase-3 into account, four main binding sites (S1–S4) are determined in which the binding to the S2 and S3-pockets are responsible for inhibitory activity and selectivity of caspase-3, respectively^8–10^. This knowledge along with the importance of this family clearly helps medicinal chemists to design new specific inhibitors of caspase enzymes^11–19^.

**Figure 1.**
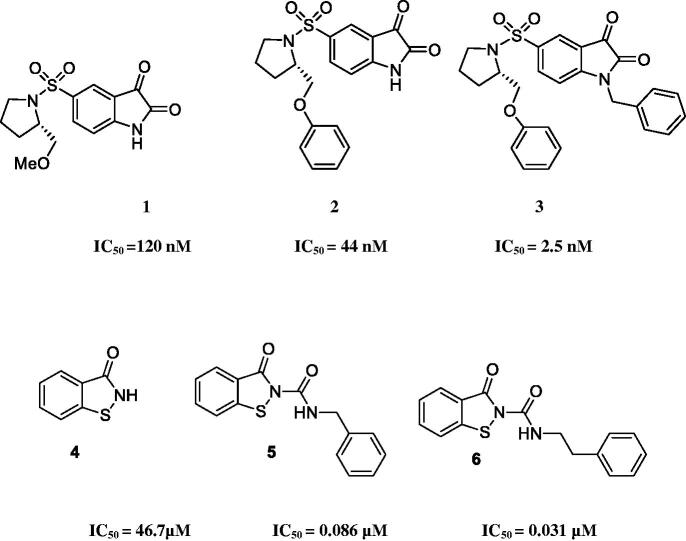
Some of the reported caspase-3 inhibitors. IC_50_ = 120 nM; IC_50_ = 44 nM; IC_50_ = 2.5 nM. IC_50_ = 46.7µM; IC_50_ = 0.086 µM; IC_50_ = 0.031 µM

Isatin sulphonamides are introduced as a new class of potent and selective non-peptide caspase-3 and −7 inhibitors. Previously, various isatin sulphonamide derivatives were prepared and evaluated as caspase-3 inhibitors^20–25^. The studies indicated the connection between carbonyl group of isatin ring and cysteine thiol in the binding site of the enzyme. 5-Pyrrolidinyl sulphonyl isatins are evidently found effective in inhibition of the caspase-3 and −7 *in vitro*. The selectivity of 5-pyrrolidinyl sulphonyl isatins is referred to the interaction of pyrrolidine ring with S2 subsite of enzyme without the interaction with S1 subsite of caspase-3^26^. The side-chains, attached to pyrrolidine meaning methoxymethyl or phenoxymethyl groups, occupy the S3 pocket. In this regard, many studies have been focussed on the synthesis of several modified isatin derivatives (**1**), relying on the structure-activity relationship (SAR) studies (Figure 1). Interestingly, it was observed that good IC_50_ values in nanomolar ranges are obtained when hydrophobic groups are attached to the N-1 position of structure **1** (Figure 1). Furthermore, the amide moiety is also found necessary in producing various potent inhibitors^27–29^. Considering the above mentioned findings about the importance of isatin sulphonamide derivatives, especially as caspase-3 inhibitors and following our ongoing projects on the design and synthesis of biologically active agents^30–37^, we synthesised isatin based compounds containing *N*-aryl acetamide and *N*-prop-2-yn-1-yl as caspase-3 and −7 inhibitors through the structural modification of compound **1**.

**Figure 2.**
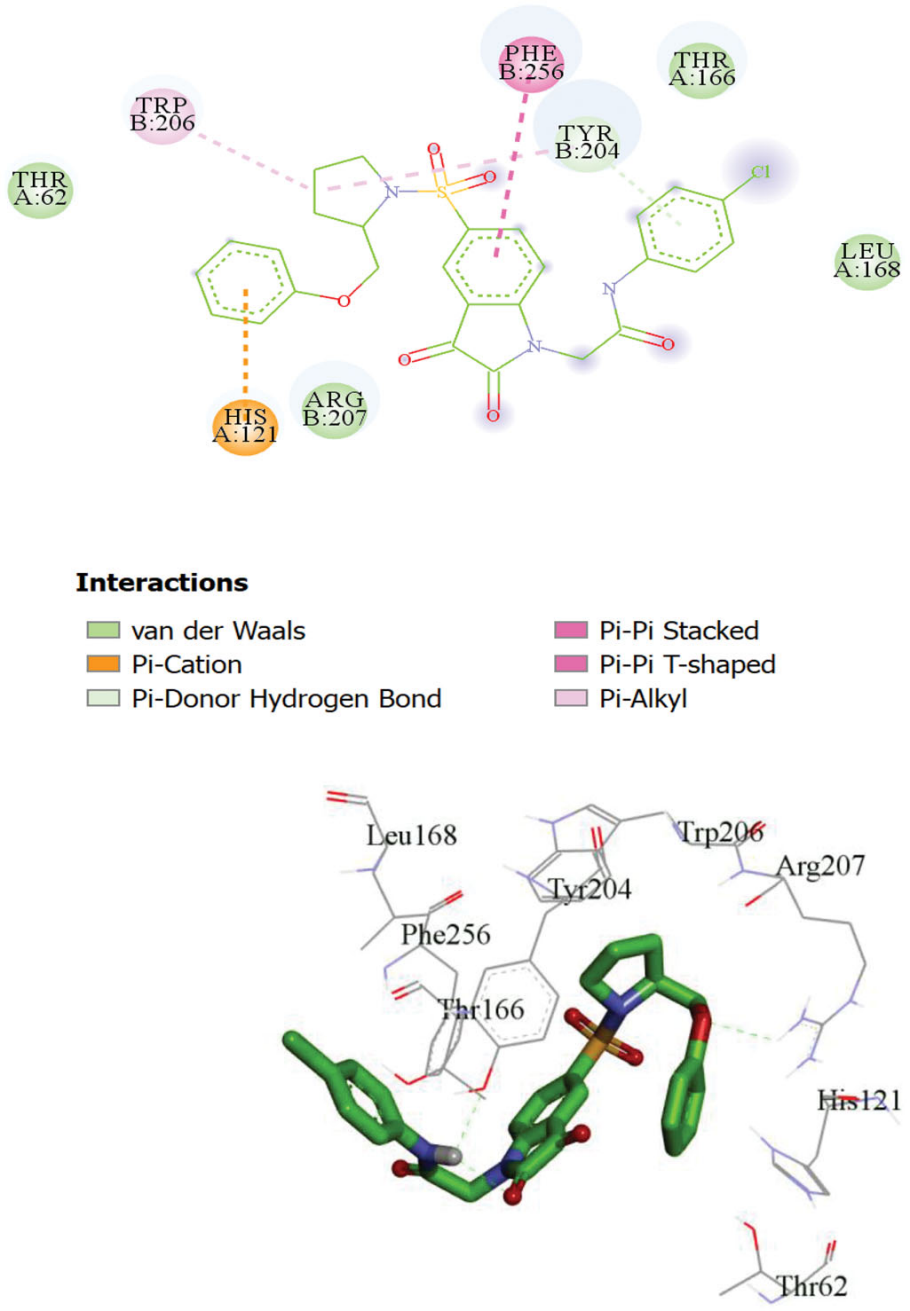
2 D and 3 D representations of **20d** interactions with caspase-3 active site.

## Results and discussion

### Chemistry

**Scheme 1.**
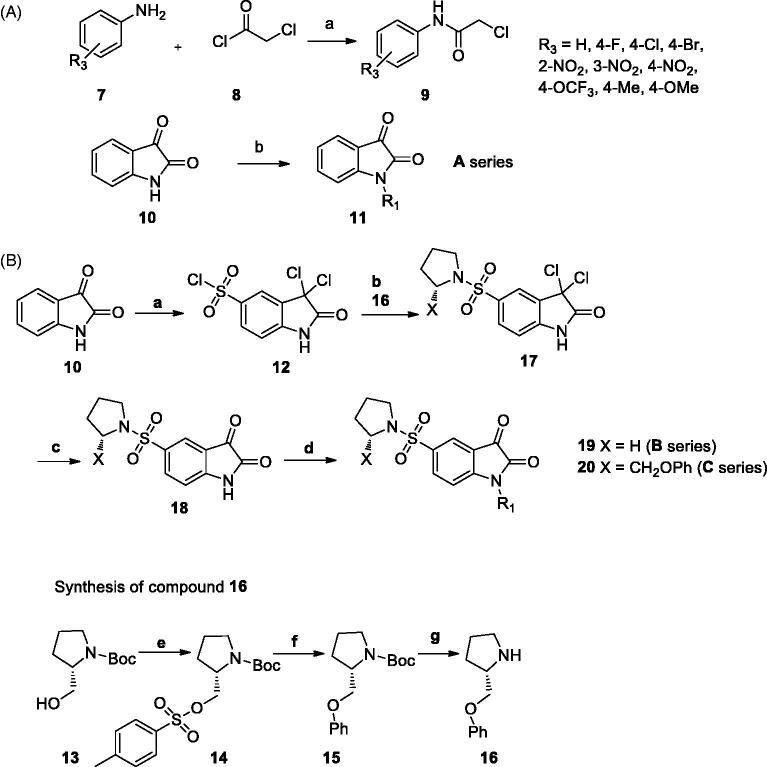
(A) Synthesis route for A series. Reagents and conditions. a: CH_2_Cl_2_, Et_3_N; b: NaH, DMF (B) Synthesis route for B series. Reagents and conditions. a: ClSO_3_H; b: pyrrolidine or **16**, Et_3_N, DMF, c: acetic acid; d: **9** or propargyl bromide, NaH, DMF, 0 ˚C; e: *p*-toluenesulfonyl chloride, pyridine; f: phenol, NaH, THF; g: TFA, CH_2_Cl_2._

First of all, the *N*-alkylated isatin derivatives (**11a–k**) were obtained in 60–85% yields from the alkylation reaction of isatin **10** with propargyl bromide or 2-chloro-*N*-arylacetamide derivatives^38–40^, synthesised from the reaction of chloroacetyl choride and aromatic amines (Part A, Scheme 1)^41^.

The synthesis of *N*-alkylated substituted 5-[1-(pyrrolidinyl)sulphonyl] isatin derivatives was started from heating isatin **10** in chlorosulfonic acid at 60 °C which is followed by amination with pyrrolidine or 2-phenoxymethyl pyrrolidine in dimethyl formamide (DMF)^42^. The subsequent hydrolysis in acetic acid and addition of 2-chloro-*N*-arylacetamides **9** or propargyl bromide led to compounds **19a–k** (64–85%) and **20a–k** (47–65%) in good yields (Part B, Scheme 1).

In this paper, 33 compounds are synthesised and their structures are deduced by IR, ^1^H, ^13 ^C NMR, mass spectroscopy, and elemental analysis. For example, the IR spectrum of these three series showed the stretching bands, related to C=O bonds of ketone and amide functional groups at nearly 1700 and 1670 cm^−1^, respectively. The mass spectrum of each compound displayed the molecular ion (M^+^) peak, which is consistent with a 1:1 adduct, formed by the substitution at NH of isatin and loss of chlorine and bromine atom of propargyl bromide or 2-chloro-*N*-arylacetamide derivatives. The ^1^H-NMR spectrum of compounds exhibited the characteristic signals at δ 4.3–4.6 and 8.2–8.8 ppm related to NCH_2_ and NH, respectively. The characteristic signals related to pyrrolidine and isatin moiety at aliphatic and aromatic region confirmed the structures of final compounds. The ^1^H-decoupled ^13 ^C-NMR spectrum of compounds showed characteristic signals at related aliphatic and aromatic regions which are in agreement with the proposed structure.

### Biological activity

The inhibitory activities of newly synthesised 2–(2,3-dioxoindolin-1-yl)-*N*-substituted phenyl acetamide, 1-(prop-2-yn-1-yl)indoline-2,3-dione and two series of compounds containing 1-(pyrrolidinyl)sulphonyl and 2-(phenoxymethyl)pyrrolidin-1-yl)sulphonyl substitution at C^5^ position of isatin core (**B, C**, Table 1) against caspase-3 and −7 were evaluated by using the acetyl-DEVD-AMC fluorogenic substrate assay. The results are expressed as inhibition percentage and IC_50_ values in Table 1. We used Ac-DEVD-CHO as the positive control.

**Table 1.** Structures of compounds **11a–k**, **19a–k**, and **20a–k** displaying inhibitory effects on caspase-3 and -7.

^a^IC_50_ values are expressed as Mean ± SD of three experiments. ^b^N.D. = Not determined. ^c^IC_50_ amount for Ac-DEVD-CHO is 0.016 ± 0.002 μM. ^d^The values given in bracket are percentage inhibition. ^d^Selectivity Index (SI) was calculated as IC_50_ caspase-7/IC_50_ caspase-3.

As can be seen in Table 1, those compounds containing no substituent at C^5^ position of isatin core (R_2_ = H) are weak inhibitors compared to the positive control. All amounts are provided as inhibition percentage at 20 μg/ml. Among this series, the best and weakest activity was observed in **11c** and **11f** with inhibition percentage of 71% and 5%, respectively. The presence of 2-(phenoxymethyl)pyrrolidine functionality on isatin core led to the more active compounds against caspase-3 and −7 than that of substituted ones with pyrrolidin-1-yl sulphonyl moiety.

In compounds **20a–k**, the comparison between the *para* substituted derivatives revealed that the electron-donating substituents (**20j**, **20k**) exhibited the lowest enzymatic inhibition. The most active compound was the 4-chlorophenylacetamide containing derivative, meaning **20d** against caspase-3 and −7. Compounds **20a–c** and **20f** have also appreciable IC_50_ values and can be regarded as moderated caspase-3 and −7 inhibitors in comparison to Ac-DEVD-CHO (IC_50_ = 0.016 ± 0.002 μM). In compounds **19a–k** and **20 a–k**, the least electronegative and more bulky atom, bromine, had clear negative effect on inhibitory potency of the compound compared to fluorine and chlorine containing derivatives. As previously reported, compounds with a selectivity index greater than 1.5 are considered as selective inhibitors of caspase-3, so, compounds **19a**, **19d**, **19e**, **20c**, **20d**, and **20e** exhibited this selectivity. Regarding the significant activity and selectivity of compound **20d**, this compound could be studied for further modification to develop novel hit compounds.

### Docking study

**Figure 3.**
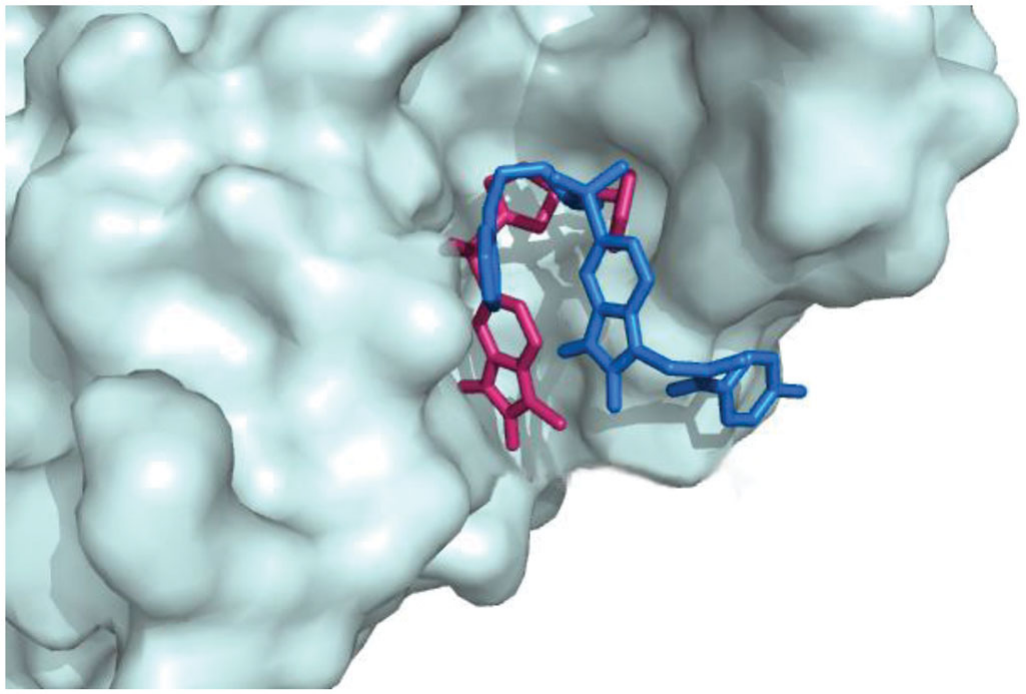
Superimposition of the binding pose for **20d** and natural ligand at the 1GFW active site.

**Figure 4.**
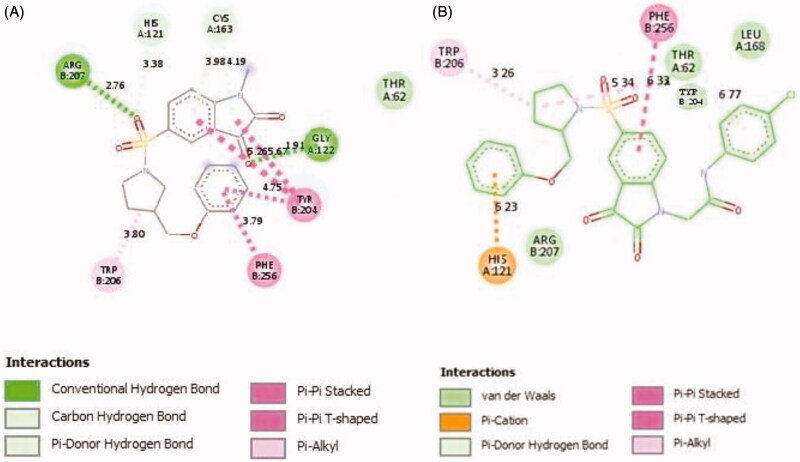
2 D representations of **20d (A)** and isatin sulphonamide (**B**) interactions with caspase-3 active site.

To investigate the binding mode of these potent inhibitors, molecular docking computations were performed using Autodock Tools (ver.1.5.6) programme^43^. Compound **20d** was docked into the active site of caspase-3 crystallographic structure (PDB ID: 1GFW), retrieved from protein data bank (http://www.rcsb.org/pdb/home/home.do) (Figure 2). The phenyl ring of phenoxymethyl group formed pi-cation interaction with HIS:121. The isatin core formed T-shaped π-π interactions with His 121 and Tyr 204. His 121 formed a carbon hydrogen bond in isatin sulphonamide crystal ligand. A Pi-alkyl interaction is formed between the oxygen of sulphonyl group and Trp 206 and Tyr 204. The carbonyl moiety interacted through π-sulfur with Cys 163 in compound **20d** and through π-hydrogen bond in isatin sulphonamide. The π-π stacked interaction is formed between isatin core and Phe 256 in isatin sulphonamide and compound **20d**. Moreover, N-phenylacetamide substitution provided enough length for more efficient interactions, like an additional van der Waals interaction between LeuA 168 and ThrA 166 and phenyl moiety. Table 2, presentesd the comparision between the type of interaction and involved amino acid residues of the most active compound, **20d**, and isatin sulphonamide. These interactions along with distances are schematically presented in Figure 3. Superimposition of the binding pose of **20d** and natural ligand at the 1GFW active site is shown in Figure 4. The binding interaction energy of compound **20d** is −4.04 kcal/mol, which stated that this compound is less potent than statin sulphonamide (-5.44 kcal/mol) towards caspase-3.

**Table 2.** The interactions of compound **20d** and natural ligand in 1GFW at the active site.

Interaction type	20d	Isatin Sulphonamide
Van der waals	–	–
Conventional hydrogen bond	–	Arg 207, Gly 122
Carbon hydrogen bond	–	His 121
Pi-pi stacked	Phe 256	Phe 256
Pi-pi T-shaped	His 121, Tyr 204	Tyr 204
Pi-alkyl	Trp 206. Tyr 204	Trp 206
Pi-cation	His 121	–
Pi-hydrogen bond	Tyr 204	Cys 163
Pi-sulfur	Cys 163	–

## Conclusion

A series of novel isatin-sulphonamide derivatives were designed, synthesised and evaluated for their caspase-3 and −7 inhibitory activity. The results showed that most of the synthesised compounds exhibited moderate inhibitory activity against caspase-3 and −7. The results revealed that 4-chloro phenylacetamide derivative **20d** exhibited the best profile of inhibitory activity on caspase-3 with IC_50_ value of 2.33 µM. The docking studies showed the perfect binding of compound **20d** to the active site of caspase-3 enzyme. The prepared product **20d** in the present study may be subjected to further optimisation to find more effective agent as caspase-3 inhibitor.

## Experimental

### Chemistry

5-[1-(Pyrrolidinyl)sulphonyl] isatin derivatives **18** were prepared by the reaction of isatin **7,** chlorosulfonic acid, pyrrolidine or 2-phenoxymethyl pyrrolidine **16** (Scheme 1). 2-Chloro-*N*-phenylacetamide derivatives **9**^38–40^ and 2-phenoxymethyl pyrrolidine **16**^22^ used in the synthesis of target products were conveniently prepared based on the previously reported procedure.

Other starting materials, chemical reagents, and solvents used in this study were commercially available (from Merck and Aldrich Chemicals) and were used without further purification. TLC was conducted on silica gel 250 micron. Melting points were determined on a Kofler hot stage apparatus and are uncorrected. The IR spectra were run on a Shimadzu 470 spectrophotometer (potassium bromide disks). Mass spectra were recorded on an Agilent Technologies (HP) 5973 mass spectrometer operating at an ionisation potential of 70 eV. The NMR spectra were recorded on a Varian unity 500 spectrometer, and the chemical shifts (δ) are reported in parts per million (ppm) relative to tetramethylsilane (TMS) as an internal standard.

#### General procedure for the N-alkylation of isatin, 5-[1-(pyrrolidinyl)sulphonyl] isatins, 5-((2-(phenoxymethyl)pyrrolidin-1-yl)sulphonyl)isatin

Sodium hydride (0.25 mmol) was added to the stirred solution of isatin **10** or intermediate **18** (0.25 mmole) in DMF (3 ml), and the reaction was continued for 15 min at 0 °C. Corresponding *N*-phenylacetamides **9** or propargyl bromide (0.25 mmol) was added and the reaction was continued for one hour. TLC was used to find reaction completion time. Water (20 ml) was added to the reaction mixture and extracted with ethyl acetate. Resulted crude product was purified over flash column chromatography (mobile phase: ethyl acetate: hexane 20:80) to yield pure products **11a–k**.

**1-(Prop-2-yn-1-yl)indoline-2,3-dione (11a)**^44^**:** White solid; Yield: 85%; m.p. 158–160 °C; IR (KBr, cm^−1^): 1718 (C=O_Ketone_), 1678 (C=O_Amide_); ^1^H-NMR (500 MHz, DMSO-d_6_): 2.90 (s, 1H, CH_Acetylene_), 4.36 (s, 2H, CH_2_), 7.22 (d, *J =* 8.5 Hz, 1H, H_7_), 7.35 (d, *J =* 8.5 Hz, 1H), 7.47 (t, *J =* 7.85 Hz, 1H), 7.52 (t, *J =* 7.85 Hz, 1H); ^13 ^C NMR (125 MHz, DMSO-*d*_6_): 37.0, 75.1, 82.0, 126.2, 126.6, 128.3, 135.1, 135.1, 145.6, 150.1, 163.3, 181.1; Anal. Calcd. For C_11_H_7_NO_2_: C, 71.35; H, 3.81; N, 7.56; Found: C, 71.07; H, 3.58; N, 7.84.

**2–(2,3-Dioxoindolin-1-yl)-*N*-phenylacetamide (11b):** White solid; Yield 78%; m.p. 174–176 °C; IR (KBr, cm^−1^): 3348 (NH), 1710 (C=O_Ketone_), 1680 (C=O_Amide_), 1660 (C=O_Amide_); ^1^H-NMR (500 MHz, DMSO-*d*_6_): 4.64 (s, 2H, CH_2_), 7.07 (d, *J* = 7.2 Hz, 1H), 7.14 (t, *J* = 7.2 Hz, 1H), 7.19 (t, *J* = 7.9 Hz, 1H), 7.32 (t, *J* = 7.9 Hz, 2H), 7.49 (d, *J* = 7.9 Hz, 2H), 7.70–7.62 (m, 2H),7.94 (s, 1H, NH); ^13 ^C-NMR (125 MHz, DMSO-*d*_6_): 43.9, 110.2, 117.9, 121.2, 125.7, 127.6, 132.7, 134.8, 145.7, 148.5, 151.6, 162.6, 166.3, 182.1; Anal. Calcd. For C_16_H_12_N_2_O_3_: C, 68.56; H, 4.32; N, 9.99; Found: C, 68.27; H, 4.04; N, 10.17; MS (*m/z*, %): 280 (M^+^, 41) 146 (100), 134 (25), 90 (57), 77 (33), 55 (76).

***2–(2,3-Dioxoindolin-1-yl)-N-(4-fluorophenyl)acetamide* (11c):** White solid; Yield 70%; m.p. 199–201 °C; IR (KBr, cm^−1^): 3340 (NH), 1700 (C=O_Ketone_), 1688 (C=O_Amide_), 1665 (C=O_Amide_); ^1^H-NMR (500 MHz, DMSO-d_6_): 4.64 (s, 2H, CH_2_), 6.89 (d, *J* = 7.5 Hz, 2H), 6.95 (d, *J* = 7.0 Hz, 1H), 6.99 (d, *J* = 7.5 Hz, 2H), 7.14 (t, *J* = 7.0 Hz, 1H), 7.58–7.63 (m, 2H), 8.05 (s, 1H, NH); ^13 ^C-NMR (125 MHz, DMSO-d_6_): 46.6, 110.6, 121.3, 125.6, 127.2, 128.3, 130.5, 134.5, 148.1, 150.9, 158.3 (*J_C-F_* = 250 Hz), 162.6, 167.0, 181.8; Anal. Calcd. For C_16_H_11_FN_2_O_3_: C, 64.43; H, 3.72; N, 9.39; Found: C, 64.67; H, 3.44; N, 9.55; MS (*m/z*, %): 298 (M^+^, 63) 146 (100), 152 (32), 96 (48), 57 (40).

***N-(4-Chlorophenyl)-2–(2,3-dioxoindolin-1-yl)acetamide* (11d):** White solid; Yield: 72%; m.p. 177–179 °C; IR (KBr, cm^−1^): 3356 (NH), 1708 (C=O_Ketone_), 1682 (C=O_Amide_), 1655 (C=O_Amide_); ^1^H-NMR (500 MHz, CDCl_3_): 4.38 (s, 2H, CH_2_), 6.99 (d, *J* = 8.0 Hz, 1H), 7.21 (d, *J* = 6.9 Hz, 2H), 7.24 (t, *J* = 8.0 Hz, 1H), 7.29 (d, *J* = 6.9 Hz, 2H), 7.61 (t, *J* = 8.0 Hz, 2H), 8.01 (s, 1H, NH). ^13 ^C-NMR (125 MHz, CDCl_3_): 43.8, 110.9, 117.7, 124.5, 125.6, 128.7, 137.3 (2 C), 138.7, 150.1, 158.5, 165.7, 182.4. Anal. Calcd. For C_16_H_11_ClN_2_O_3_: C, 61.06; H, 3.52; N, 8.90; Found: C, 61.37; H, 3.74; N, 9.05; MS (*m/z*, %): 316 (M + 2^+^, 39), 314 (M^+^, 14) 168 (35), 146 (100), 152 (52), 112 (64), 90 (28), 56 (40).

***N-(4-Bromophenyl)-2–(2,3-dioxoindolin-1-yl)acetamide* (11e):** White solid; Yield: 66%; m.p. 179–181 °C; IR (KBr, cm^−1^): 3330 (NH), 1698 (C=O_Ketone_), 1670 (C=O_Amide_), 1656 (C=O_Amide_); ^1^H-NMR (500 MHz, DMSO-d_6_): 4.49 (s, 2H, CH_2_), 6.90 (d, *J* = 7.0 Hz, 1H), 6.90 (d, *J* = 7.0 Hz, 1H), 7.08 (d, *J* = 7.5 Hz, 2H), 7.16–7.21 (m, 1H), 7.54–7.56 (m, 2H), 7.64 (d, *J* = 7.9 Hz, 2H), 8.00 (s, 1H, NH). ^13 ^C-NMR (125 MHz, DMSO-d_6_): 45.0, 110.6, 115.4, 117.2, 118.3, 124.9, 126.8, 135.8, 138.0, 149.8, 150.4, 158.3, 163.3, 182.1, Anal. Calcd. For C_16_H_11_BrN_2_O_3_: C, 53.50; H, 3.09; N, 7.80; Found: C, 53.87; H, 3.44; N, 7.57; MS (*m/z*, %): 359 (M + 2^+^, 26), 357 (M^+^, 24), 211 (47), 154 (69), 146 (100), 90 (36), 56 (50).

***2–(2,3-Dioxoindolin-1-yl)-N-(2-nitrophenyl)acetamide* (11f):** White solid; Yield: 77%; m.p. 186–188 °C; IR (KBr, cm^−1^): 3340 (NH), 1725 (C=O _Ketone_), 1685 (C=O _Amide_), 1665 (C=O_Amide_); ^1^H-NMR (500 MHz, CDCl_3_): 4.58 (s, 2H, CH_2_), 7.00 (d, *J* = 7.0 Hz, 1H), 7.11 (t, *J* = 7.0 Hz, 1H), 7.27–7.33 (m, 2H), 7.37 (d, *J* = 8.0 Hz, 1H), 7.58 (t, *J* = 7.0 Hz, 1H), 7.80 (t, *J* = 8.0 Hz, 1H), 7.92 (d, *J* = 8.0 Hz, 1H), 8.50 (s, 1H, NH). ^13 ^C-NMR (125 MHz, CDCl_3_): 45.8, 123.6, 127.3, 129.4, 129.9, 129.9, 132.3, 134.8, 135.2, 136.4, 139.9, 145.6, 146.5, 151.0, 161.2, 168.4, 179.3. Anal. Calcd. For C_16_H_11_N_3_O_5_: C, 59.08; H, 3.41; N, 12.92; Found: C, 59.37; H, 3.14; N, 13.17; MS (*m/z*, %): 325 (M^+^, 48), 179 (51), 146 (100), 123 (44), 92 (29), 57 (42).

***2–(2,3-Dioxoindolin-1-yl)-N-(3-nitrophenyl)acetamide* (11g):** White solid; Yield: 75%; m.p. 191–192 °C; IR (KBr, cm^−1^): 3348 (NH), 1725 (C=O_Ketone_), 1680 (C=O_Amide_), 1658 (C=O_Amide_); ^1^H-NMR (500 MHz, CDCl_3_): 4.49 (s, 2H, CH_2_), 7.08–7.11 (m, 1H), 7.24 (d, *J* = 8.0 Hz, 1H), 7.32–7.34 (m, 1H), 7.56–7.58 (m, 1H), 7.64 (d, *J* = 7.0 Hz, 1H), 7.83 (t, *J* = 7.0 Hz, 1H), 8.00 (s, 1H), 8.18 (d, *J* = 7.5 Hz, 1H), 8.39 (s, 1H, NH). ^13 ^C-NMR (125 MHz, CDCl_3_): 45.60, 124.1, 127.8, 127.9, 130.0, 130.2, 132.2, 135.4, 135.9, 139.4, 140.3, 146.0, 146.9, 160.4, 168.6, 179.6. Anal. Calcd. For C_16_H_11_N_3_O_5_: C, 59.08; H, 3.41; N, 12.92; Found: C, 59.35; H, 3.73; N, 13.19.

***2–(2,3-Dioxoindolin-1-yl)-N-(4-nitrophenyl)acetamide* (11h):** White solid; Yield: 69%; m.p. 179–181 °C; IR (KBr, cm^−1^): 3338 (NH), 1718 (C=O_Ketone_), 1680 (C=O _Amide_), 1665 (C=O_Amide_); ^1^H-NMR (500 MHz, DMSO-d_6_): 4.49 (s, 2H, CH_2_), 7.13 (d, *J* = 7.2 Hz, 1H), 7.19–7.24 (m, 1H), 7.38–7.41 (m, 2H), 7.66–7.69 (m, 1H), 7.85–7.88 (m, 1H), 8.00–8.04 (m, 2H), 8.37 (s, 1H, NH). ^13 ^C-NMR (125 MHz, DMSO-d_6_): 46.1, 124.6, 127.3, 128.1, 129.1, 131.5, 135.6, 139.1, 145.4, 145.7, 150.4, 161.6, 167.0, 178.4. Anal. Calcd. For C_16_H_11_N_3_O_5_: C, 59.08; H, 3.41; N, 12.92; Found: C, 59.31; H, 3.79; N, 13.11.

***2–(2,3-Dioxoindolin-1-yl)-N-(4-(trifluoromethoxy)phenyl)acetamide* (11i):** White solid; Yield: 75%; m.p. 209– 211 °C; IR (KBr, cm^−1^): 3348 (NH), 1721 (C=O_Ketone_), 1680 (C=O_Amide_), 1665 (C=O_Amide_); ^1^H-NMR (500 MHz, DMSO-d_6_): 4.52 (s, 2H, CH_2_), 7.23–7.27 (m, 3H), 7.11 (d, *J* = 7.5 Hz, 1H), 7.38 (d, *J* = 8.5 Hz, 2H), 7.57 (t, *J* = 7.5 Hz, 1H), 7.93 (d, *J* = 7.5 Hz, 1H), 8.35 (s, 1H, NH). ^13 ^C-NMR (125 MHz, DMSO-d_6_): 45.2, 116.0, 121.6, 124.5, 126.5, 128.3, 128.6, 131.7, 134.2, 135.4, 138.9, 149.2, 161.9, 166.7, 178.9. Anal. Calcd. For C_17_H_11_F_3_N_2_O_4_: C, 56.05; H, 3.04; N, 7.69; Found: C, 55.91; H, 3.42; N, 7.84; MS (*m/z*, %): 364 (M^+^, 26), 218 (M^+^, 18), 162 (44), 146 (100), 90 (51), 56 (32).

***2–(2,3-Dioxoindolin-1-yl)-N-(p-tolyl)acetamide* (11j):** White solid; Yield: 60%; m.p. 196–198 °C; IR (KBr, cm^−1^): 3354 (NH), 1728 (C=O_Ketone_), 1688 (C=O _Amide_), 1659 (C=O_Amide_); ^1^H-NMR (500 MHz, DMSO-d_6_): 2.34 (s, 3H), 4.59 (s, 2H, CH_2_), 6.97 (d, *J* = 7.6 Hz, 1H), 7.21 (d, *J* = 8.0 Hz, 2H), 7.27 (d, *J* = 7.6 Hz, 1H), 7.41 (d, *J* = 8.0 Hz, 2H), 7.71 (d, *J* = 7.8 Hz, 1H), 7.88 (t, *J* = 7.8 Hz, 1H), 8.37 (s, 1H, NH). Anal. Calcd. For C_17_H_14_N_2_O_3_: C, 69.38; H, 4.79; N, 9.52; Found: C, 69.51; H, 4.92; N, 9.66; MS (*m/z*, %): 294 (M^+^, 42), 148 (34), 146 (100), 91 (44), 56 (68).

***2–(2,3-Dioxoindolin-1-yl)-N-(4-methoxyphenyl)acetamide* (11k):** White solid; Yield: 68%; m.p. 201–203 °C; IR (KBr, cm^−1^): 3354 (NH), 1728(C=O_Ketone_), 1688 (C=O_Amide_), 1659 (C=O_Amide_); ^1^H-NMR (500 MHz, DMSO-d_6_): 3.70 (s, 3H, OMe), 4.47 (s, 2H, CH_2_), 6.88 (d, *J* = 8.0 Hz, 2H), 7.08–7.11 (m, 1H), 7.20–7.25 (m, 3H), 7.55–7.57 (m, 1H), 7.91–7.92 (m, 1H), 8.22 (s, 1H, NH). ^13 ^C-NMR (125 MHz, DMSO-d_6_): 45.9, 55.0, 113.8, 116.1, 121.6, 124.4, 126.5, 128.4, 134.2, 149.0, 149.2, 161.9 (2 C), 167.6, 178.4. Anal. Calcd. For C_17_H_14_N_2_O_4_: C, 65.80; H, 4.55; N, 9.03; Found: C, 65.55; H, 4.28; N, 9.33; MS (*m/z*, %): 310 (M^+^, 26), 164 (18), 108 (44), 146 (100), 92 (51), 58 (32).

***1-(Prop-2-yn-1-yl)-5-(pyrrolidin-1-ylsulfonyl)indoline-2,3-dione* (19a):** White solid; Yield: 82%; m.p. 203–205 °C; IR (KBr, cm^−1^): 3345 (NH), 1710 (C=O_Ketone_), 1680 (C=O_Amide_), 1660 (C=O_Amide_); ^1^H-NMR (500 MHz, DMSO-d_6_): 1.63–1.70 (m, 4H, CH_2-Pyrrole_), 2.98–3.04 (m, 4H, CH_2-Pyrrole_), 3.24 (s, 1H, CH), 4.73 (s, 2H, CH_2_), 7.13 (d, *J* = 8.5 Hz, 1H), 7.82 (d, *J* = 8.5 Hz, 1H), 8.08 (s, 1H); ^13 ^C-NMR (125 MHz, DMSO-d_6_): 25.9, 47.2, 61.9, 71.9, 73.8, 112.0, 126.5, 128.0, 136.6, 152.5, 161.5, 166.6, 179.8; Anal. Calcd. For C_15_H_14_N_2_O_4_S: C, 56.59; H, 4.43; N, 8.80; Found: C, 56.77; H, 4.11; N, 8.57; MS (*m/z*, %): 318 (M^+^, 42), 279 (26), 208 (51), 146 (100), 71 (22).

***2–(2,3-Dioxo-5-(pyrrolidin-1-ylsulfonyl)indolin-1-yl)-N-phenylacetamide* (19b):** White solid; Yield: 85%; m.p. 238–240 °C; IR (KBr, cm^−1^): 3364 (NH), 1725 (C=O_Ketone_), 1684 (C=O_Amide_), 1655 (C=O_Amide_); ^1^H-NMR (500 MHz, DMSO-d_6_): 1.60– 1.67 (m, 4H, CH_2-Pyrrole_), 3.08–3.12 (m, 4H, CH_2-Pyrrole_), 4.49 (s, 2H, CH_2_), 6.92 (d, *J =* 7.5 Hz, 1H), 7.12 (d, *J =* 8.8 Hz, 2H), 7.36 (d, *J =* 8.0 Hz, 1H), 7.44 (t, *J =* 7.5 Hz, 1H), 7.64 (t, *J =* 8.0 Hz, 2H), 7.96 (s, 1H), 8.48 (s, 1H, NH); ^13 ^C-NMR (125 MHz, DMSO-d_6_): 23.8, 47.8, 62.2, 123.7, 129.0, 129.2, 131.5, 134.4, 142.6, 143.2, 143.4, 145.4, 147.2, 162.3, 167.2, 182.3; Anal. Calcd. For C_20_H_19_N_3_O_5_S: C, 58.10; H, 4.63; N, 10.16; Found: C, 58.37; H, 4.91; N, 10.35; MS (*m/z*, %): 413 (M^+^, 39), 279 (43), 146 (100), 134 (57), 71 (29).

***2–(2,3-Dioxo-5-(pyrrolidin-1-ylsulfonyl)indolin-1-yl)-N-(4-fluorophenyl)acetamide* (19c):** White solid; Yield: 80%; m.p. 191–193 °C; IR (KBr, cm^−1^): 3345 (NH), 1711 (C=O_Ketone_), 1670 (C=O_Amide_), 1654 (C=O_Amide_); ^1^H-NMR (500 MHz, DMSO-d_6_): 1.69–1.77 (m, 4H, CH_2-Pyrrole_) 2.98–3.11 (m, 4H, CH_2-Pyrrole_), 4.41 (s, 2H, CH_2_), 4.41 (s, 2H, CH_2_), 6.94 (d, *J =* 7.5 Hz, 1H), 7.14 (d, *J =* 8.8 Hz, 2H), 7.36 (d, *J =* 8.8 Hz, 2H), 7.42 (d, *J =* 7.5 Hz, 1H), 8.05 (s, 1H), 8.49 (s, 1H, NH); ^13 ^C-NMR (125 MHz, DMSO-d_6_): 25.5, 48.0, 60.0, 110.5, 114.4 (*J*_C-F_ = 6.75 Hz), 119.2, 126.8 (*J*_C-F_ = 24.5 Hz), 128.1, 131.0, 135.6, 149.0, 151.6, 158.6, 162.0 (*J*_C–F_ = 245 Hz), 165.6, 183.2; Anal. Calcd. For C_20_H_18_FN_3_O_5_S: C, 55.68; H, 4.21; N, 9.74; Found: C, 55.37; H, 4.51; N, 9.49; MS (*m/z*, %): 431 (M^+^, 27), 278 (39), 154 (51), 146 (100), 135 (57), 95 (31).

***N-(4-Chlorophenyl)-2–(2,3-dioxo-5-(pyrrolidin-1-ylsulfonyl)indolin-1-yl)acetamide* (19d):** White solid; Yield: 71%; m.p. 300–302 °C; IR (KBr, cm^−1^): 3330 (NH), 1706 (C=O_Ketone_), 1685 (C=O_Amide_), 1658 (C=O_Amide_); ^1^H-NMR (500 MHz, DMSO-d_6_): 1.65–1.58 (m, 4H, CH_2-Pyrrole_). 2.98–3.05 (m, 4H, CH_2-Pyrrole_), 4.24 (s, 2H, CH_2_), 6.77 (d, *J =* 8.65 Hz, 1H), 7.38 (d, *J =* 8.0 Hz, 2H), 7.65 (d, *J =* 8.0 Hz, 2H), 7.72 (d, *J =* 8.65 Hz, 1H), 8.08 (s, 1H), 8.34 (s, 1H, NH); ^13 ^C-NMR (125 MHz, DMSO-d_6_): 26.7, 47.5, 62.8, 111.2, 120.3, 125.9, 126.8, 128.4, 129.3, 129.6, 134.9, 144.5, 151.4, 158.4, 165.9, 182.8; Anal. Calcd. For C_20_H_18_ClN_3_O_5_S: C, 53.63; H, 4.05; N, 9.38; Found: C, 53.85; H, 3.78; N, 9.09; MS (*m/z*, %): 449 (M + 2^+^, 36), 447 (M^+^, 11), 276 (43), 168 (35), 146 (100), 134 (18), 110 (29).

***N-(4-Bromophenyl)-2–(2,3-dioxo-5-(pyrrolidin-1-ylsulfonyl)indolin-1-yl)acetamide* (19e):** White solid; Yield: 68%; m.p. 290–292 °C; IR (KBr, cm^−1^): 3334 (NH), 1705 (C=O_Ketone_), 1675 (C=O_Amide_), 1660 (C=O_Amide_); ^1^H-NMR (500 MHz, DMSO-d_6_): 1.62–1.66 (m, 4H, CH_2-Pyrrole_) 3.00–3.07 (m, 4H, CH_2-Pyrrole_), 4.49 (s, 2H, CH_2_), 7.11 (d, *J =* 8.6 Hz, 1H), 7.49 (d, *J =* 8.5 Hz, 2H), 7.61 (d, *J =* 8.5 Hz, 2H), 7.78 (d, *J =* 7.5 Hz, 1H), 8.18 (s, 1H), 8.52 (s, 1H, NH); ^13 ^C-NMR (125 MHz, DMSO-d_6_): 20.7, 43.8, 62.7, 113.3, 123.2, 125.9, 126.0, 127.0, 127.4, 128.5, 129.1, 130.7142.5, 160.8, 165.5, 181.8; Anal. Calcd. For C_20_H_18_BrN_3_O_5_S: C, 48.79; H, 3.69; N, 8.53; Found: C, 48.45; H, 3.46; N, 8.78; MS (*m/z*, %): 493 (M + 2^+^, 28), 491 (M^+^, 26), 278 (51), 211 (29), 154 (48), 146 (100), 133 (24).

***2–(2,3-Dioxo-5-(pyrrolidin-1-ylsulfonyl)indolin-1-yl)-N-(2-nitrophenyl)acetamide* (19f):** White solid; Yield: 74%; m.p. 195–197 °C; IR (KBr, cm^−1^): 3345 (NH), 1725 (C=O_Ketone_), 1685 (C=O_Amide_), 1660 (C=O_Amide_); ^1^H-NMR (500 MHz, DMSO-d_6_): 1.61–1.66 (m, 4H, CH_2-Pyrrole_), 2.99–3.07 (m, 4H, CH_2-Pyrrole_), 4.49 (s, 2H, CH_2_), 7.12 (d, *J =* 7.0 Hz, 1H), 7.24 (t, *J =* 8.0 Hz, 1H), 7.52 (d, *J =* 8.6 Hz, 1H), 7.73 (t, *J =* 8.0 Hz, 1H), 8.01 (s, 1H), 8.12 (t, *J =* 7.0 Hz, 1H), 8.37 (d, *J =* 7.0 Hz, 1H), 8.66 (s, 1H, NH); Anal. Calcd. For C_20_H_18_N_4_O_7_S: C, 52.40; H, 3.96; N, 12.22; Found: C, 52.53; H, 4.09; N, 12.35; MS (*m/z*, %): 458 (M^+^, 41), 279 (37), 211 (40), 163 (27), 146 (100), 135 (19).

***2–(2,3-Dioxo-5-(pyrrolidin-1-ylsulfonyl)indolin-1-yl)-N-(3-nitrophenyl)acetamide* (19g):** White solid; Yield 73%; m.p. 223–225 °C; IR (KBr, cm^−1^): 3340 (NH), 1720 (C=O_Ketone_), 1680 (C=O_Amide_), 1660 (C=O_Amide_); ^1^H-NMR (500 MHz, DMSO-d_6_): 1.58–1.65 (m, 4H, CH_2-Pyrrole_), 2.89–3.03 (m, 4H, CH_2-Pyrrole_), 4.38 (s, 2H, CH_2_), 7.12 (d, *J =* 7.5 Hz, 1H), 7.32 (t, *J =* 7.5 Hz, 1H), 7.71 (d, *J =* 7.5 Hz, 1H), 7.82 (d, *J =* 7.5 Hz, 1H), 8.01 (s, 1H), 8.17 (s, 1H), 8.20 (s, 1H), 8.54 (s, 1H, NH); ^13 ^C-NMR (125 MHz, DMSO-d_6_): 25.1, 49.5, 61.3, 110.1, 122.8, 123.4, 126.1, 128.2, 129.0, 130.4, 132.1, 134.1, 135.0, 143.4, 150.9, 158.9, 167.6, 180.0; Anal. Calcd. For C_20_H_18_N_4_O_7_S: C, 52.40; H, 3.96; N, 12.22; Found: C, 52.78; H, 4.14; N, 11.90.

***2–(2,3-Dioxo-5-(pyrrolidin-1-ylsulfonyl)indolin-1-yl)-N-(4-nitrophenyl)acetamide* (19h):** White solid; Yield: 69%; m.p. 216–218 °C; IR (KBr, cm^−1^): 3340 (NH), 1728 (C=O_Ketone_), 1680 (C=O_Amide_), 1646 (C=O_Amide_); ^1^H-NMR (500 MHz, DMSO-d_6_): 1.64–1.67 (m, 4H, CH_2-Pyrrole_), 2.94–2.98 (m, 4H, CH_2-Pyrrole_), 4.68 (s, 2H, CH_2_), 7.12 (d, *J =* 7.5 Hz, 1H), 7.46 (dd, *J =* 8.0, 3.5 Hz, 2H), 7.68 (d, *J =* 7.5 Hz, 1H), 7.90 (s, 1H), 8.21 (dd, *J =* 8.0 Hz, *J =* 3.5 Hz, 2H), 8.51 (s, 1H, NH); ^13 ^C-NMR (125 MHz, DMSO-d_6_): 25.2, 48.1, 60.3, 111.3, 122.9, 123.4, 130.4, 132.1, 133.2, 134.1, 142.4, 143.3, 149.2, 160.1, 167.6, 183.4; Anal. Calcd. For C_20_H_18_N_4_O_7_S: C, 52.40; H, 3.96; N, 12.22; Found: C, 52.67; H, 4.23; N, 12.53.

***2–(2,3-Dioxo-5-(pyrrolidin-1-ylsulfonyl)indolin-1-yl)-N-(4-(trifluoromethoxy)phenyl)acetamide* (19i):** White solid; Yield: 64%; m.p. 266–267 °C; IR (KBr, cm^−1^): 3340 (NH), 1710 (C=O_Ketone_), 1678 (C=O_Amide_), 1656 (C=O_Amide_); ^1^H-NMR (500 MHz, DMSO-d_6_): 1.65–1.70 (m, 4H, CH_2-Pyrrole_), 3.08–3.13- (m, 4H, CH_2-Pyrrole_), 4.66 (s, 2H, CH_2_), 6.85 (d, *J =* 7.0 Hz, 2H), 7.14 (d, *J =* 8.5 Hz, 1H), 7.20 (d, *J =* 7.0 Hz, 2H), 7.44 (d, *J =* 8.5 Hz, 1H), 8.04 (s, 1H), 8.54 (s, 1H, NH); ^13 ^C-NMR (125 MHz, DMSO-d_6_): 25.8, 47.4, 62.5, 113.1, 123.8, 129.0, 129.2, 131.5, 134.4, 142.6, 143.2, 143.4, 147.2, 151.4, 160.2, 167.3, 180.9; Anal. Calcd. For C_21_H_18_F_3_N_3_O_6_S: C, 50.70; H, 3.65; N, 8.45; Found: C, 50.47; H, 3.81; N, 8.12; MS (*m/z*, %): 497 (M^+^, 51), 279 (41), 218 (23), 161 (29), 146 (100), 77 (22).

***2–(2,3-Dioxo-5-(pyrrolidin-1-ylsulfonyl)indolin-1-yl)-N-(p-tolyl)acetamide* (19j):** White solid; Yield: 77%; m.p. 248–250 °C; IR (KBr, cm^−1^): 3356 (NH), 1716 (C=O_Ketone_), 1688 (C=O_Amide_), 1653 (C=O_Amide_); ^1^H-NMR (500 MHz, DMSO-d_6_): 1.64–1.69 (m, 4H, CH_2-Pyrrole_), 2.25 (s, 3H, CH_3_), 3.09–3.17 (m, 4H, CH_2-Pyrrole_), 4.64 (s, 2H, CH_2_), 7.12 (d, *J =* 7.85 Hz, 2H), 7.39 (d, *J =* 8.25 Hz, 1H), 7.43 (d, *J =* 7.85 Hz, 2H), 7.86 (s, 1H), 8.11 (d, *J =* 8.25 Hz, 1H), 8.47 (s, 1H, NH); ^13 ^C-NMR (125 MHz, DMSO-d_6_):18.3, 24.9, 49.4, 59.9, 112.0, 119.2, 126.9, 127.5, 128.9, 129.4, 130.6, 131.6, 134.3, 148.7, 158.2, 165.1, 183.3; Anal. Calcd. For C_21_H_21_N_3_O_5_S: C, 59.00; H, 4.95; N, 9.83; Found: C, 59.37; H, 5.21; N, 10.12; MS (*m/z*, %): 427 (M^+^, 52), 278 (41), 150 (29), 146 (100), 134 (39), 57 (36).

***2–(2,3-Dioxo-5-(pyrrolidin-1-ylsulfonyl)indolin-1-yl)-N-(4-methoxyphenyl)acetamide* (19k):** White solid; Yield: 73%; m.p. 194–196 °C; IR (KBr, cm^−1^): 3356 (NH), 1716 (C=O_Ketone_), 1688 (C=O_Amide_), 1653 (C=O_Amide_); ^1^H-NMR (500 MHz, DMSO-d_6_): 1.64–1.68 (m, 4H, CH_2-Pyrrole_), 2.82 (t, *J =* 6.0 Hz, 4H, CH_2-Pyrrole_), 3.94 (s, 3H, O-CH_3_), 4.19 (s, 2H, CH_2_), 6.85 (d, *J =* 8.0 Hz, 2H), 7.10 (d, *J =* 8.5 Hz, 1H), 7.41 (d, *J =* 8.0 Hz, 2H), 7.50 (d, *J =* 8.5 Hz, 1H), 8.12 (s, 1H), 8.46 (s, 1H, NH);^13^C-NMR (125 MHz, DMSO-d_6_): 25.1, 45.7, 55.3, 63.7, 110.3, 114.5, 122.4 (2 C), 123.4, 126.5, 129.7, 132.1, 134.0, 159.1, 160.2, 167.6, 181.1; Anal. Calcd. For C_21_H_21_N_3_O_6_S: C, 56.88; H, 4.77; N, 9.48; Found: C, 56.57; H, 4.31; N, 9.12; MS (*m/z*, %): 443 (M^+^, 52), 278 (41), 150 (29), 146 (100), 134 (39), 57 (36).

***(S)-5-((2-(Phenoxymethyl)pyrrolidin-1-yl)sulphonyl)-1-(prop-2-yn-1-yl)indoline-2,3-dione* (20a):** White solid; Yield: 65%; m.p. 282–284 °C; IR (KBr, cm^−1^): 1725 (C=O_Ketone_), 1680 (C=O_Amide_), 1658 (C=O_Amide_); ^1^H-NMR (500 MHz, DMSO-d_6_): 1.61–1.67 (m, 4H, CH_2-Pyrrole_), 2.66–2.69 (m, 2H, CH_2-Pyrrole_), 3.38–3.42 (m, 2H, CH_Acetylene_,CH_Pyrrole_), 4.00–4.09 (m, 2H, O-CH_2_), 4.61 (s, 2H, N-CH_2_), 6.93 (d, *J =* 7.2 Hz, 2H), 7.28 (t, *J =* 7.2 Hz, 3H), 7.42 (d, *J =* 7.7 Hz, 1H), 7.87 (s, 1H), 8.21 (d, *J =* 7.7 Hz, 1H); ^13 ^C-NMR (125 MHz, DMSO-d_6_): 23.6, 26.0, 46.1, 58.5, 62.4, 72.0, 73.6, 118.6, 123.7, 124.4, 126.2, 128.5, 129.8, 138.5, 139.5, 151.9, 161.9, 164.0, 179.6; Anal. Calcd. For C_22_H_20_N_2_O_5_S: C, 62.25; H, 4.75; N, 6.60; Found: C, 62.51; H, 4.97; N, 6.32; MS (*m/z*, %): 424 (M^+^, 48), 385 (31), 248 (54), 208 (40), 176 (23), 146 (100), 107 (21), 77 (30).

***(S)-2–(2,3-Dioxo-5-((2-(phenoxymethyl)pyrrolidin-1-yl)sulphonyl)indolin-1-yl)-N-phenylacetamide* (20b):** White solid; Yield: 56%; m.p. 238–240 °C; IR (KBr, cm^−1^): 1720 (C=O_Ketone_), 1688 (C=O_Amide_), 1660 (C=O_Amide_); ^1^H-NMR (500 MHz, DMSO-d_6_): 1.78–1.84 (m, 4H, CH_2-Pyrrole_), 2.62–2.65 (m, 2H, CH_2-Pyrrole_), 3.30–3.36 (m, 1H, CH_Chiral_), 3.87 (d, *J =* 12.0 Hz, 1H, O-CH_2-Diastropic_), 4.14 (d, *J =* 12.0 Hz, 1H, O-CH_2-Diastropic_), 4.60 (s, 2H, N-CH_2_), 6.99–7.05 (m, 3H), 7.37–7.43 (m, 6H), 7.50 (d, *J =* 8.0 Hz, 2H), 8.02 (d, *J =* 8.0 Hz, 1H), 8.10 (s, 1H), 8.46 (s, 1H, NH); ^13 ^C-NMR (125 MHz, DMSO-d_6_): 22.5, 27.6, 45.6, 58.5, 63.2, 117.7, 119.2, 120.9, 127.2, 128.4, 128.7, 128.9, 129.2, 129.5, 134.0, 135.2, 135.7, 139.8, 151.1, 161.3, 166.2, 179.7; Anal. Calcd. For C_27_H_25_N_3_O_6_S: C, 62.42; H, 4.85; N, 8.09; Found: C, 62.66; H, 4.97; N, 8.39; MS (*m/z*, %): 519 (M^+^, 52), 278 (41), 150 (29), 146 (100), 134 (39), 77 (36).

***(S)-2–(2,3-Dioxo-5-((2-(phenoxymethyl)pyrrolidin-1-yl)sulphonyl)indolin-1-yl)-N-(4-fluorophenyl)acetamide* (20c):** White solid; Yield: 53%; m.p. 108–110 °C; IR (KBr, cm^−1^): 1725 (C=O_Ketone_), 1690 (C=O_Amide_), 1655 (C=O_Amide_); ^1^H-NMR (500 MHz, DMSO-d_6_): 1.78–1.84 (m, 4H, CH_2-Pyrrole_), 2.62–2.65 (m, 2H, CH_2-Pyrrole_), 3.43–3.46 (m, 1H, CH_Chiral_), 3.73 (d, *J =* 13.0 Hz, 1H, O-CH_2-Diastropic_), 4.10 (d, *J =* 13.0 Hz, 1H, O-CH_2-Diastropic_), 4.58 (s, 2H, N-CH_2_), 6.95–7.02 (m, 3H), 7.27 (t, *J =* 7.0 Hz, 2H), 7.35 (d, *J =* 7.0 Hz, 2H), 7.41 (d, *J =* 7.0 Hz, 1H), 7.59 (t, *J =* 7.0 Hz, 2H), 8.02 (d, *J =* 8.0 Hz, 1H), 8.08 (s, 1H), 8.56 (s, 1H, NH); ^13 ^C-NMR (125 MHz, DMSO-d_6_): 22.4, 27.6, 45.6, 57.3, 62.4, 117.5, 117.7, 119.2, 120.9, 127.2, 128.4, 130.1, 131.4, 131.6, 135.7, 136.9, 142.4, 151.5, 160.9, 161.6, 163.5, 181.2; Anal. Calcd. For C_27_H_24_FN_3_O_6_S: C, 60.33; H, 4.50; N, 7.82; Found: C, 60.05; H, 4.82; N, 7.55; MS (*m/z*, %): 537 (M^+^, 41), 385 (28), 152 (35), 240 (49), 146 (100), 97 (26), 77 (29).

***(S)-N-(4-chlorophenyl)-2–(2,3-dioxo-5-((2-(phenoxymethyl)pyrrolidin-1-yl)sulphonyl)indolin-1-yl)acetamide* (20d):** White solid; Yield: 48%; m.p. 209–211 °C; IR (KBr, cm^−1^): 1718 (C=O_Ketone_), 1700 (C=O_Amide_), 1670 (C=O_Amide_); ^1^H-NMR (500 MHz, DMSO-d_6_): 1.78–1.84 (m, 4H, CH_2-Pyrrole_), 2.61–2.63 (m, 2H, CH_2-Pyrrole_), 3.44–3.48 (m, 1H, CH_Chiral_), 3.99 (d, *J =* 13.0 Hz, 1H, O-CH_2-Diastropic_), 4.18 (d, *J =* 13.0 Hz, 1H, O-CH_2-Diastropic_), 4.58 (s, 2H, N-CH_2_), 7.03 (t, *J =* 7.0 Hz, 2H), 7.34 (d, *J =* 7.5 Hz, 1H), 7.36–7.41 (m, 3H), 7.48–7.55 (m, 4H), 8.03 (d, *J =* 8.0 Hz, 1H), 8.07 (s, 1H),8.58 (s, 1H, NH); ^13 ^C-NMR (125 MHz, DMSO-d_6_): 22.5, 28.6, 46.6, 28.6, 62.4, 117.3, 119.2, 127.2, 128.3, 129.0, 129.2, 130.8, 131.0, 132.8, 134.2, 135.2, 135.7, 139.9, 152.0, 161.7, 166.5, 180.8; Anal. Calcd. For C_27_H_24_ClN_3_O_6_S: C, 58.53; H, 4.37; N, 7.58; Found: C, 58.73; H, 4.18; N, 7.29; MS (*m/z*, %): 554 (M^+^, 26), 385 (62), 240 (48), 168 (21), 146 (100), 112 (17), 77 (41), 58 (55).

***(S)-N-(4-Bromophenyl)-2–(2,3-dioxo-5-((2-(phenoxymethyl)pyrrolidin-1-yl)sulphonyl)indolin-1-yl)acetamide* (20e):** White solid; Yield: 51%; m.p. 196–198 °C; IR (KBr, cm^−1^): 1722 (C=O_Ketone_), 1680 (C=O_Amide_), 1660 (C=O_Amide_); ^1^H-NMR (500 MHz, DMSO-d_6_): 1.78–1.83 (m, 4H, CH_2-Pyrrole_), 2.60–2.63 (m, 2H, CH_2-Pyrrole_), 3.40–3.45 (m, 1H, CH_Chiral_), 4.13 (d, *J =* 13.0 Hz, 1H, O-CH_2-Diastropic_), 4.28 (d, *J =* 13.0 Hz, 1H, O-CH_2-Diastropic_), 4.68 (s, 2H, N-CH_2_), 7.03 (t, *J =* 7.0 Hz, 2H), 7.16–7.21 (m, 3H), 7.37–7.42 (m, 5H), 8.02 (d, *J =* 8.0 Hz, 1H), 8.14 (s, 1H), 8.56 (s, 1H, NH); ^13 ^C-NMR (125 MHz, DMSO-d_6_): 22.6, 28.6, 46.6, 58.1, 62.4, 116.0, 117.5, 117.7, 119.0, 127.2, 128.7, 129.0, 129.6, 130.9, 135.2, 135.7, 139.0, 139.8, 150.0, 162.3, 167.8, 179.0; Anal. Calcd. For C_27_H_24_BrN_3_O_6_S: C, 54.19; H, 4.04; N, 7.02; Found: C, 54.34; H, 4.44; N, 7.38; MS (*m/z*, %): 600 (M + 2^+^, 37), 598 (M^+^, 35), 385 (41), 240 (29), 155 (42), 146 (100), 77 (36).

***(S)-2–(2,3-Dioxo-5-((2-(phenoxymethyl)pyrrolidin-1-yl)sulphonyl)indolin-1-yl)-N-(2-nitrophenyl)acetamide* (20f):** White solid; Yield: 47%; m.p. 180–182 °C; IR (KBr, cm^−1^): 1720 (C=O_Ketone_), 1685 (C=O_Amide_), 1654 (C=O_Amide_); ^1^H-NMR (500 MHz, DMSO-d_6_): 1.79–1.83 (m, 4H, CH_2-Pyrrole_), 2.63–2.67 (m, 2H, CH_2-Pyrrole_), 3.38–3.41 (m, 1H, CH_Chiral_), 3.86 (d, *J =* 13.0 Hz, 1H, O-CH_2-Diastropic_), 4.17 (d, *J =* 13.0 Hz, 1H, O-CH_2-Diastropic_), 4.68 (s, 2H, N-CH_2_), 6.99–7.04 (m, 3H), 7.28 (t, *J =* 8.0 Hz, 1H), 7.34 (t, *J =* 7.0 Hz, 2H), 7.38–7.41 (m, 2H), 7.77 (t, *J =* 8.0 Hz, 1H), 8.03 (d, *J =* 8.0 Hz, 1H), 8.07 (d, *J =* 7.5 Hz, 1H), 8.18 (s, 1H), 8.62 (s, 1H, NH); ^13 ^C-NMR (125 MHz, DMSO-d_6_): 22.6, 28.6, 46.6, 58.1, 62.4, 116.0, 117.5, 117.9, 119.2, 120.9, 127.2, 128.3, 130.1, 131.4, 131.6, 135.2, 135.8, 140.0, 142.4, 143.6, 144.0, 161.1, 167.0, 179.3; Anal. Calcd. For C_27_H_24_N_4_O_8_S: C, 57.44; H, 4.28; N, 9.92; Found: C, 57.74; H, 4.49; N, 10.21; MS (*m/z*, %): 564 (M^+^, 48), 324 (39), 385 (53), 240 (27), 179 (33), 144 (100), 123 (61), 77 (25).

***(S)-2–(2,3-Dioxo-5-((2-(phenoxymethyl)pyrrolidin-1-yl)sulphonyl)indolin-1-yl)-N-(3-nitrophenyl)acetamide* (20g):** White solid; Yield: 52%; m.p. 181–183 °C; IR (KBr, cm^−1^): 1725 (C=O_Ketone_), 1686 (C=O_Amide_), 1656 (C=O_Amide_); ^1^H-NMR (500 MHz, DMSO-d_6_): 1.78–1.87 (m, 4H, CH_2-Pyrrole_), 2.63–2.66 (m, 2H, CH_2-Pyrrole_), 3.49–3.52 (m, 1H, CH_Chiral_), 3.77–3.80 (m, 1H, O-CH_2-Diastropic_), 3.97–4.01 (m, 1H, O-CH_2-Diastropic_), 4.58 (s, 2H, N-CH_2_), 7.00–7.05 (m, 3H), 7.36 (d, *J =* 7.5 Hz, 1H), 7.41 (d, *J =* 7.5 Hz, 1H), 7.42–7.44 (m, 1H), 7.45 (d, *J =* 7.0 Hz, 2H), 7.48 (t, *J =* 8.0 Hz, 1H), 8.03 (d, *J =* 8.0 Hz, 1H), 8.07 (d, *J =* 7.0 Hz, 1H), 8.18 (s, 1H), 8.71 (s, 1H, NH); ^13 ^C-NMR (125 MHz, DMSO-d_6_): 22.52, 26.61, 47.25, 57.36, 62.40, 117.5, 117.8, 122.8, 127.2, 128.5, 129.9, 130.9 (2 C), 131.7, 131.9, 135.1, 136.2, 139.9, 143.8, 144.5, 151.9, 162.4, 166.7, 179.1; Anal. Calcd. For C_27_H_24_N_4_O_8_S: C, 57.44; H, 4.28; N, 9.92; Found: C, 57.71; H, 4.46; N, 10.17.

***(S)-2–(2,3-Dioxo-5-((2-(phenoxymethyl)pyrrolidin-1-yl)sulphonyl)indolin-1-yl)-N-(4-nitrophenyl)acetamide* (20h):** White solid; Yield: 51%; m.p. 162–164 °C; IR (KBr, cm^−1^): 1718 (C=O_Ketone_), 1688 (C=O_Amide_), 1655 (C=O_Amide_); ^1^H-NMR (500 MHz, DMSO-d_6_): 1.78–1.82 (m, 4H, CH_2-Pyrrole_), 2.59–2.63 (m, 2H, CH_2-Pyrrole_), 3.44–3.47 (m, 1H, CH_Chiral_), 3.86–3.89 (m, 1H, O-CH_2-Diastropic_), 4.06–4.10 (m, 1H, O-CH_2-Diastropic_), 4.59 (s, 2H, N-CH_2_), 6.98–7.02 (m, 3H), 7.35–7.53 (m, 5H), 8.13–8.15 (m, 1H), 8.15–8.18 (m, 2H), 8.20 (s, 1H), 8.62 (s, 1H, NH); ^13 ^C-NMR (125 MHz, DMSO-d_6_): 22.5, 27.0, 45.5, 57.1, 61.3, 117.5, 117.8, 127.2, 128.2, 129.9, 130.9, 131.7, 131.9, 135.1, 135.7, 139.9, 142.5, 144.1, 150.7, 161.9, 166.8, 180.0; Anal. Calcd. For C_27_H_24_N_4_O_8_S: C, 57.44; H, 4.28; N, 9.92; Found: C, 57.77; H, 4.52; N, 9.66.

***(S)-2–(2,3-Dioxo-5-((2-(phenoxymethyl)pyrrolidin-1-yl)sulphonyl)indolin-1-yl)-N-(4-(trifluoromethoxy)phenyl)acetamide* (20i):** White solid; Yield: 56%; m.p. 218–220 °C; IR (KBr, cm^−1^): 1724 (C=O_Ketone_), 1690 (C=O_Amide_), 1668 (C=O_Amide_); ^1^H-NMR (500 MHz, DMSO-d_6_): 1.80–1.84 (m, 4H, CH_2-Pyrrole_), 2.60–2.68 (m, 2H, CH_2-Pyrrole_), 3.70–3.73 (m, 1H, CH_Chiral_), 3.90 (d, *J =* 13.0 Hz, 1H, O-CH_2-Diastropic_), 4.14 (d, *J =* 13.0 Hz, 1H, O-CH_2-Diastropic_), 4.57 (s, 2H, N-CH_2_), 7.03 (t, *J =* 7.5 Hz, 2H), 7.32 (t, *J =* 7.5 Hz, 1H), 7.43–7.47 (m, 3H), 7.53 (d, *J =* 7.0 Hz, 1H), 7.59 (d, *J =* 8.0 Hz, 2H), 7.72 (d, *J =* 8.0 Hz, 1H), 7.92 (d, *J =* 7.0 Hz, 1H), 8.10 (s, 1H), 8.90 (s, 1H, NH);^13^C-NMR (125 MHz, DMSO-d_6_): 22.5, 27.8, 44.8, 57.2, 61.4, 117.6, 119.1, 120.8, 125.3, 127.2, 127.7, 128.9, 129.3, 131.0, 133.5, 135.3, 136.2 (2 C), 144.7, 150.8, 162.0, 166.5, 179.0; Anal. Calcd. For C_28_H_24_F_3_N_3_O_7_S: C, 55.72; H, 4.01; N, 6.96; Found: C, 55.89; H, 4.25; N, 7.15; MS (*m/z*, %): 603 (M^+^, 60), 442 (54), 385 (29), 240 (52), 161 (42), 146 (100), 93 (17), 77 (29).

***(S)-2–(2,3-Dioxo-5-((2-(phenoxymethyl)pyrrolidin-1-yl)sulphonyl)indolin-1-yl)-N-(p-tolyl)acetamide* (20j):** White solid; Yield: 48%; m.p. 209–211 °C; IR (KBr, cm^−1^): 1723 (C=O_Ketone_), 1677 (C=O_Amide_), 1649 (C=O_Amide_); ^1^H-NMR (500 MHz, DMSO-d_6_): 1.76–1.81 (m, 4H, CH_2-Pyrrole_), 2.09 (s, 3H), 2.59–2.62 (m, 2H, CH_2-Pyrrole_), 3.44–3.48 (m, 1H, CH_Chiral_), 3.79 (d, *J =* 13.0 Hz, 1H, O-CH_2-Diastropic_), 4.10 (d, *J =* 13.0 Hz, 1H, O-CH_2-Diastropic_), 4.51 (s, 2H, N-CH_2_), 7.01–7.04 (m, 3H), 7.13 (d, *J =* 7.0 Hz, 1H), 7.23 (d, *J =* 7.5 Hz, 2H), 7.34 (t, *J =* 7.5 Hz, 2H), 7.40 (d, *J =* 7.0 Hz, 2H), 8.12 (d, *J =* 7.5 Hz, 1H), 8.19 (s, 1H), 8.50 (s, 1H, NH); ^13 ^C-NMR (125 MHz, DMSO-d_6_): 18.6, 24.5, 28.2, 45.5, 57.9, 61.4, 117.8, 119.2, 120.7, 126.5, 127.2, 128.2, 129.4, 130.8, 131.5, 135.1, 135.7, 137.0, 139.9, 151.5, 162.6, 166.9, 179.8; Anal. Calcd. For C_28_H_27_N_3_O_6_S: C, 63.03; H, 5.10; N, 7.87; Found: C, 63.38; H, 5.35; N, 8.13; MS (*m/z*, %): 533 (M^+^, 52), 385 (42), 240 (38), 148 (100), 93 (37), 77 (49).

***(S)-2–(2,3-Dioxo-5-((2-(phenoxymethyl)pyrrolidin-1-yl)sulphonyl)indolin-1-yl)-N-(4-methoxyphenyl)acetamide* (20k):** White solid; Yield: 48%; m.p. 204–206 °C; IR (KBr, cm^−1^): 1720 (C=O_Ketone_), 1680 (C=O_Amide_), 1656 (C=O_Amide_); ^1^H-NMR (500 MHz, DMSO-d_6_): 1.78–1.83 (m, 4H, CH_2-Pyrrole_), 2.60–2.62 (m, 2H, CH_2-Pyrrole_), 3.46–3.50 (m, 1H, CH_Chiral_), 3.62 (s, 3H), 4.14–4.21 (m, 2H, O-CH_2_), 4.64 (s, 2H, N-CH_2_), 6.90 (d, *J =* 7.5 Hz, 2H), 7.00–7.03 (m, 3H), 7.27–7.31 (m, 2H), 7.41 (d, *J =* 7.8 Hz, 1H), 7.54 (d, *J =* 7.5 Hz, 2H), 8.08 (d, *J =* 7.8 Hz, 1H), 8.18 (s, 1H), 8.61 (s, 1H, NH); ^13 ^C-NMR (125 MHz, DMSO-d_6_): 22.5, 28.5, 46.6, 55.1, 58.3, 62.6, 109.5, 110.1, 119.3, 121.0, 127.2, 128.4, 130.2, 131.4, 131.5, 135.2, 135.8, 140.0, 142.6, 152.1, 158.5, 161.5, 163.4,179.3; Anal. Calcd. For C_28_H_27_N_3_O_7_S: C, 61.19; H, 4.95; N, 7.65; Found: C, 61.38; H, 5.12; N, 7.82; MS (*m/z*, %): 549 (M^+^, 40), 385 (21), 164 (37), 146 (100), 108 (42), 77 (61).

### Caspase-3 and -7 inhibition assay

The activity assay of caspase-3 was performed in a system of 50 µL containing 150 mM NaCl, 1 mM EDTA, 2 mM DTT, 50 mM HEPES pH 7.4, 10 µM Ac-DEVD-AMC (Bachem Bioscience, Philadelphia, PA, USA) and 2 nM caspase-3 in the 1 µL DMSO. Caspase-3 was incubated with synthesised compounds in 384-well plates for 10 min. The %inhibition of target compounds was measured at 20 µg/ml. The enzymatic activity of the caspase-3 was measured based on production of a fluorogenic substrate, 7-amino-4-methyl coumarin, which was monitored for 10 min and detected using an EnVision (PerkinElmer, Wellesley, MA, USA) at λ_ex_ = 360 nm and λ_em_= 460 nm. The initial rate of hydrolysis was determined using the early linear region of the enzymatic reaction curve. For IC_50_ determination, about 8 concentrations of the synthesised compounds were freshly prepared by three-fold serial dilutions DMSO and the assay buffer such that following the addition of the inhibitors, DMSO concentration would equal to 0.2%. and GraphPad Prism 5 software was used to calculate the IC_50_ values.

## Computational studies

Docking procedure was performed *via* Autodock Tools (1.5.6). The crystallographic structure of human caspase-3 complexed with isatin sulphonamide (PDB ID: 1GFW) were retrieved from the Protein Data Bank. The co-crystallized ligand and water molecules were eliminated and the protein was converted to the pdbqt format using Autodock Tools (1.5.6). Compounds structures were drawn and 3 D-optimized using Marvin Sketch 15.8.10, 2015, ChemAxon (http://www.chemaxon.com), then converted to pdbqt by Autodock Tools. Each docking system were completed by 50 runs and the grid box parameters were set as follows: size_x = 50; size_y = 50; size_z = 50; centred on co-ligand’s position in PDB complex. Other parameters of Autodock search by the Lamarckian genetic algorithm (LGA) were left as default except population size and maximum number of evaluations which were changed to 100 and 1000000, respectively. Finally, interactions of the compounds were illustrated by discovery studio visualiser ver.4.5 to investigate their binding mode. Docking validation were confirmed through re-docking of 1GFW co-ligand into the receptor with the same docking parameters of the compounds.
